# Effect of Laser Radiation on the Dynamics of Active Brownian Macroparticles in an Extended Plasma-Dust Monolayer

**DOI:** 10.3390/molecules26226974

**Published:** 2021-11-18

**Authors:** Ilnaz Izailovich Fairushin, Mikhail Mikhailovich Vasiliev, Oleg Fedorovich Petrov

**Affiliations:** 1 Joint Institute for High Temperatures, Russian Academy of Sciences, 125412 Moscow, Russia; mixxy@mail.ru (M.M.V.); ofpetrov@ihed.ras.ru (O.F.P.); 2 Department of Computational Physics, Institute of Physics, Kazan Federal University, 420008 Kazan, Russia

**Keywords:** dusty plasma monolayer, metal coating, thermophoretic force, Brownian motion, active matter

## Abstract

Using the modified method of Brownian dynamics, the dynamics of macroparticles with a uniform metal coating in a plasma-dust monolayer under the action of laser radiation was simulated. The time dependences of the root-mean-square and average linear displacements of particles were calculated for different initial effective parameters of nonideality and different intensities of laser radiation. A relationship was established that connects the effective parameter of nonideality of the dusty plasma system of active particles with the maximum value of the mean linear displacement of particles.

## 1. Introduction

A dusty plasma monolayer is an actively investigated physical object with a number of unique properties [1,2,3,4,5,6,7,8,9,10,11]. The kinetic temperature of the macroparticles in the dusty plasma monolayer is much higher than the temperature of the surrounding heavy particles (neutrals and plasma ions) [6]. This is due to the fact that the presence of a charge in the particles leads to the transfer of the electric energy of the gas discharge into the kinetic energy of the chaotic motion of particles in the plane of the monolayer. In this connection, the dusty plasma system can be considered as one of the options for the implementation of active matter [12,13,14,15,16,17]. In recent years, the interest in this form of matter on the part of scientists from various fields of science is growing rapidly [14]. The unusual properties of active matter are largely associated with the thermodynamic openness of such systems and, as a consequence, its study is important for solving fundamental problems of physics, chemistry and biology. The active substance is also of interest for many applications, from efficient drug delivery to the creation of “living” materials with structures and functions that cannot be achieved in passive materials [14]. In this work, the goal is to simulate the dynamics of charged macroparticles with a uniform metal coating in a plasma-dust monolayer under the action of laser radiation.

The effect of increasing the kinetic energy of particles in a dusty plasma system using laser radiation has advantages over the traditional method of heating and melting a dusty plasma crystal by reducing the pressure in the discharge chamber. In the first case, the particle charge remains practically unchanged, and the vibration amplitude of the particles in the direction perpendicular to the plane of the monolayer does not increase, the system remains stable.

In [16], the behavior of a single such particle in an RF capacitive discharge under the action of laser radiation on it was experimentally studied. It was found that such a particle performs cyclic movements along a trajectory close to a circle, and the radius of the trajectory depends on the power of the acting laser radiation. The more power, the larger the radius of the trajectory. In [17], experimental studies of the microscopic dynamics of particles of melamine-formaldehyde with a metal coating in a plasma-dust monolayer when exposed to a limited area of the system of laser radiation were presented. Under the action of laser radiation in the wavelength range below the red border of the photoelectric effect, the particle surface was heated. The collision of neutral atoms with a more heated particle is accompanied by a greater transfer of momentum than in a collision with a less heated one. Thus, a dust particle with a heated surface receives an additional external energy source to increase its own kinetic energy. The additional force associated with particle heating, as well as the Langevin force, is characterized by a random change in both magnitude and direction. However, unlike the Langevin force, this additional force is not related to the equilibrium ambient temperature. Therefore, to set it in modeling, it is necessary to use a non-standard approach, which is described below.

## 2. Statement of the Problem and Details of Modeling

Let us consider the case when a dusty plasma monolayer consists of several hundred micron polymer particles coated with a metal film. Then, the movement of each individual particle will largely depend on the interaction with neighboring particles and on external conditions. The strong interaction of particles is due to the presence of an electric charge on them and the properties of the surrounding nonequilibrium low-pressure gas discharge plasma [1,2,3,4,5,6,7,8,9,10,11]. The model assumes that the particles have the same size and composition and are uniformly covered with a thin metal film capable of absorbing laser radiation. The initial arrangement of the particles is uniform in the plane of the monolayer. The potential of Yukawa was chosen as the potential for interaction:

(1) $$\varphi \left(r\right)=\frac{Z_{d}}{r}exp\left(-\frac{r}{\lambda_{D}}\right)$$

where *Z_d_*—particle charge, *r*—the distance between two neighboring particles, *λ_D_*—Debye shielding radius, which was assumed to be equal to the mean distance between particles. *l_p_* corresponds to the parameters of the simulation of such systems and is close to actual experimental data [1]. One of the key parameters of the dusty subsystem is the effective nonideality parameter Γ*, which is determined from the following relation [18,19]:

$$\Gamma *=\frac{Z_{d}^{2}\;\left(1+\kappa +\frac{\kappa^{2}}{2}\right)}{l_{p}k_{B}T}exp\left(-\kappa \right),$$

where $\kappa =l_{p}/\lambda_{D}$—screening parameter, *T* is the kinetic temperature of the dust subsystem. The phase transition in the “crystal-liquid” system corresponds to the value Γ* ≈ 104 [19].

In addition, the particles are affected by the friction force on neutrals -$\;m\gamma {\dot{r}}_{i}$ and the determining random force, the Langevin thermostat $L_{i}$. *m* is the mass of the particle, the coefficient of friction *γ* is determined from the following expression:

$$\gamma =\delta \frac{4\pi }{3}\frac{m_{a}}{m}\;n_{a}c_{a}r_{p}.$$

Here, *δ* is the coefficient depending on the microscopic mechanism of collision of a gas atom with the particle surface, *n_a_* is the concentration of buffer gas atoms, *m_a_* is the mass of buffer gas atoms, *c_a_* is the velocity of buffer gas atoms, *r_p_* is the radius of a dusty macroparticle. For the coefficient *δ*, a value of 1.22 was chosen, which corresponds to an intermediate value between the ideal reflecting surface at *δ* = 1 and the surface of the ideal heat insulator at *δ* = 1.442 [20]. Note that in this case, the force$\;L_{i}$ is specified as a normal random variable with variance $\sqrt{2k_{B}T/\gamma dt}$, where dt is the simulation time step. It is assumed that the forces acting in the vertical direction counterbalance each other. The system is kept within the considered region by specifying potential walls at the boundaries; in the equation of motion term is responsible $F_{conf}$—the force from the border of the area (confinement). In the case under consideration, the potential trap is specified by applying mirror boundary conditions. In addition, the particles are affected by a random force $F_{i}^{tp}$, associated with the effect of thermophoresis under the heating effect of laser radiation on the metal surface of the particle, which is proportional to the density of the light flux [17]. The heated surface of the particle leads to the fact that neutral atoms of the buffer gas transfer a greater momentum upon collision with a particle than upon collision with a “cold” particle. In our case, it is assumed that the system is uniformly illuminated by laser radiation, so that the surface of all particles is simultaneously and uniformly heated. Thus, this force is responsible for the activity of the particles. In this work, the magnitude and direction of this force is set randomly with uniformly distributed values of the modulus and angle of rotation of the force vector, and the maximum value of the force is selected so as to provide the desired effective parameter of the nonideality of the dust subsystem, since an increase in force causes an increase in the effective temperature of the dust subsystem. The modulus of its average value is further denoted as $\bar{F}$. Thus, the equations of motion of particles in the system are as follows:

(2) $$m{\ddot{r}}_{i}=-Z_{d}\sum \nabla \varphi -m\gamma {\dot{r}}_{i}+L_{i}+F_{conf}+F_{i}^{tp}$$

**Figure 1:**
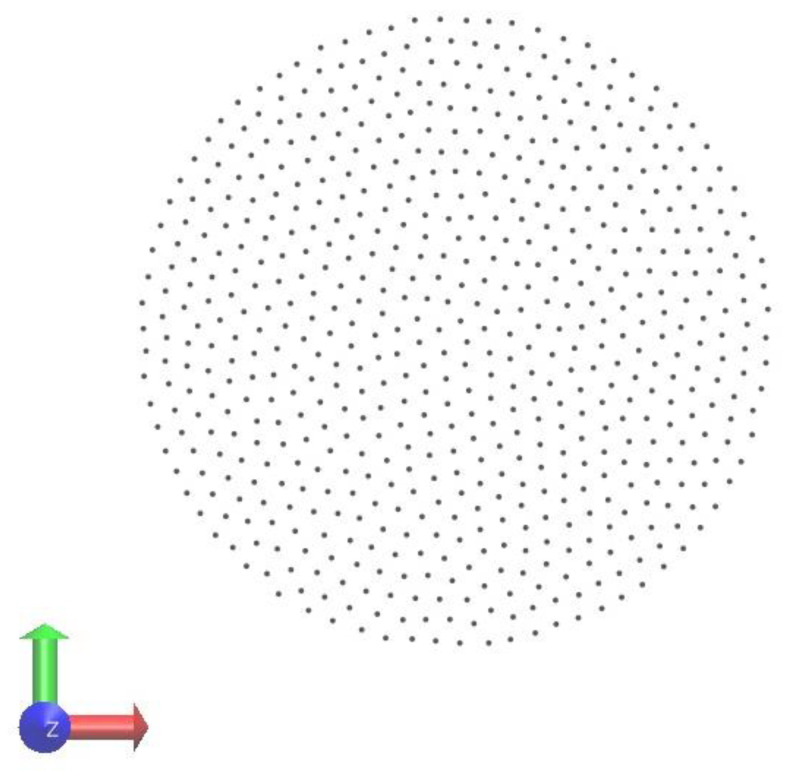
The initial configuration of the system at Γ*_0_ = 300.

**Figure 2:**
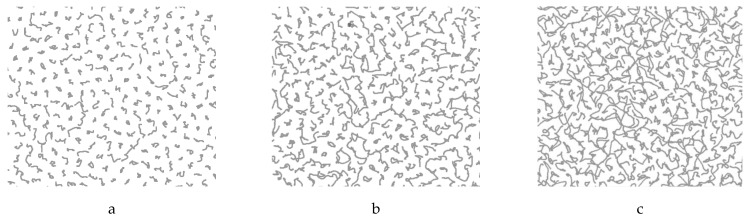
Particle trajectories in 0.3 s. Initial Γ*_0_ = 300, (**a**) $\bar{F}$ = 10 *fN*; (**b**) $\bar{F}$ = 20 *fN*; (**c**) $\bar{F}$ = 40 *fN*.

The two-dimensional region, limited by a potential trap with a radius of 28 mm (Figure 1), was filled with particles in the amount of *N* = 625, with an interparticle distance of 1 mm and a particle diameter of 10 µm, the mass of particles was calculated based on the density of melamine-formaldehyde and the thickness of the copper coating of 100 nm and amounted to 10^−9^ g. The charge on the particles was taken as equal to 10,000 electron charges, which corresponds to the data of many works, see, for example, [5]. The determination of the effective nonideality parameter Γ* was carried out by calculating the radial distribution function of particles (pair correlation function) using the method described in [21]. The studies were carried out for different values of the initial effective nonideality parameter Γ*_0_, as well as for different values of the average force $\bar{F}$ caused by the heating of the particle surface by laser radiation. After “switching on” the force $\bar{F}$, the system was held for 50 s in order to come to a stationary state, which was characterized by a fairly stable average kinetic energy of the dust subsystem. Figure 2 shows the trajectories of particles obtained in 0.3 s for different values of the force $\bar{F}$ and the initial effective parameter of nonideality Γ*_0_ = 300. As expected, with an increase in the force $\bar{F}$, an increase in the intensity of the kinetic motion of the particles (effective temperature) of the system is observed. In this case, if $\bar{F}$= 10 fN, we have Γ* ≈ 200, if $\bar{F}$ = 20 fN, we have Γ* ≈ 50, and if $\bar{F}$ = 40 fN, we have Γ* ≈ 15.

Based on the results of numerical experiments, the time dependences of the root-mean-square displacements and average linear displacements of particles along the vectors of the initial velocities of the particles were calculated.

## 3. Obtained Data, Their Analysis and Discussion

**Figure 3:**
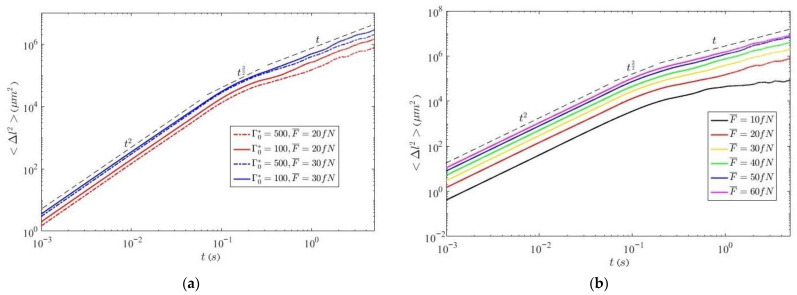
(**a**) Mean square displacements of particles at Γ*_0_ = 100 и 500, and with different values of thermophoretic force $\bar{F}$. (**b**) Mean square displacements of particles at the initial value of the effective parameter of nonideality Γ*_0_ = 500 and various values of thermophoretic force .

Figure 3a,b show the time dependences of the root-mean-square displacements of particles with a metal coating in a plasma-dust monolayer at different initial effective parameters of nonideality and different values of the average thermophoretic force $\bar{F}$.

As can be seen from these figures, the graph of the rms displacement at a higher average thermophoretic force is higher than at a lower one, which is obviously associated with a more intense kinetic motion of particles in the first case. The values of the rms displacement at a higher value of the initial effective parameter of nonideality are higher, which is due to the fact that at low Γ*_0_, the effect of the external force will manifest itself less because of the high initial average kinetic energy of the particles. It can be seen that under the action of a thermophoretic force on a dusty system for the root-mean-square displacement of particles in such a system, there are regions corresponding to the ballistic, transient, and diffusion regimes. Moreover, the transient regime is more pronounced at the minimum value of the acting thermophoretic force.

**Figure 4:**
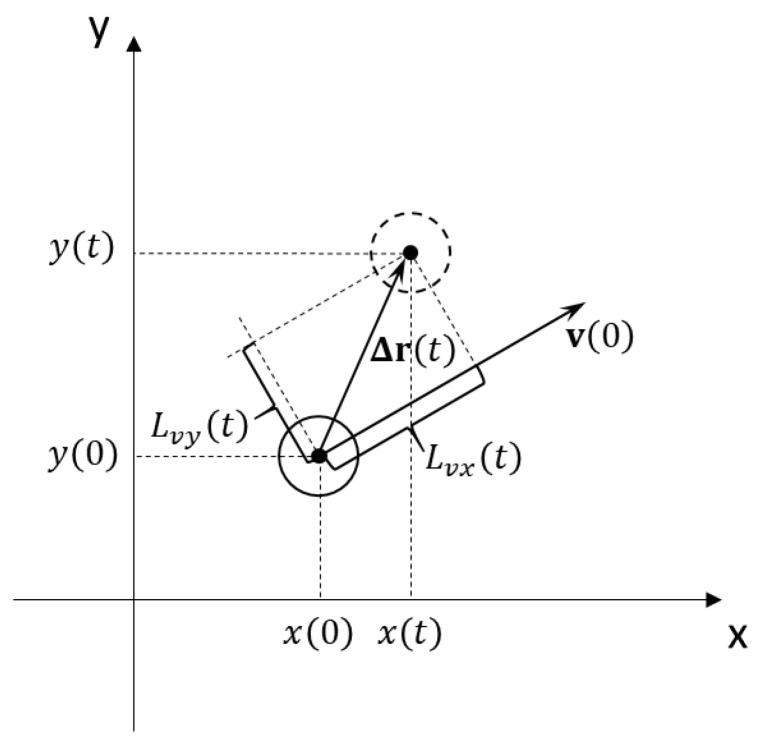
Scheme for calculating the linear displacement of a particle along and perpendicular to the direction of the initial velocity.

To characterize the active motion of a single particle, the value of the average linear displacement along the vector of the initial particle velocity [14] (Figure 4) can be used. It is noted in [14] that for a single active particle, the value of the linear displacement along the initial orientation of the particles is always nonzero.

In the case of considering a certain ensemble of particles, it is necessary to use averaging over particles, and therefore, this value is calculated using the following formula:

(3) $$\langle L_{vx}\left(t\right)\rangle =\frac{1}{N}\overset{N}{\underset{i}{\sum }}\frac{\Delta r_{i}\left(t\right)\cdot v_{i}\left(0\right)}{\left|v_{i}\left(0\right)\right|}.$$

In this case, for the average linear displacement perpendicular to the direction of the initial velocities, we have the relation:

(4) $$\langle L_{vy}\left(t\right)\rangle =\frac{1}{N}\overset{N}{\underset{i}{\sum }}\frac{\left|\Delta r_{i}\left(t\right)\times v_{i}\left(0\right)\right|}{\left|v_{i}\left(0\right)\right|}.$$

In expressions (3) and (4), the initial velocities **v*_i_***(0) are taken to be those velocities that the particles acquire 50 s after the “switching on” of the force $\bar{F}$.

**Figure 5:**
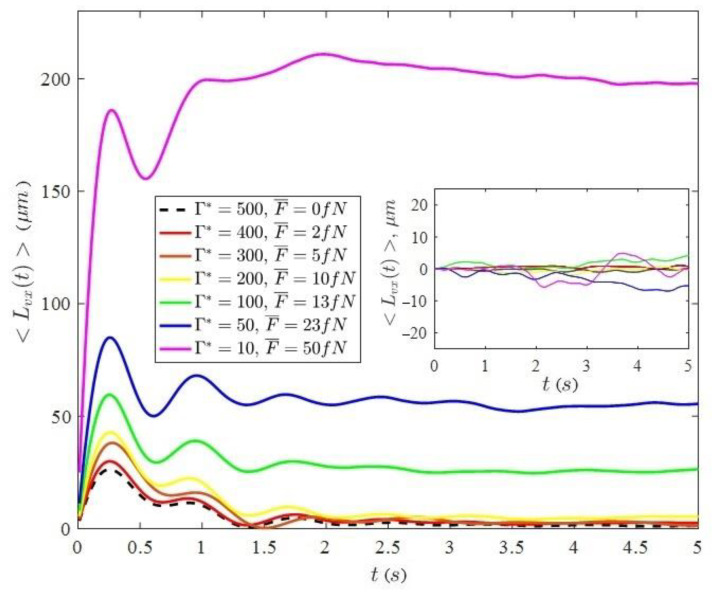
Average linear displacements along the vector of initial velocities for particles with a metal coating in a plasma-dust monolayer at different power levels of the acting laser radiation.

Figure 5 shows the graphs of the time dependences of the value $\langle L_{vx}\left(t\right)\rangle$ (on the inset of the value $\langle L_{vy}\left(t\right)\rangle$).

As can be seen from the graphs presented, the value of the average linear displacement along the vector of initial velocities first increases sharply, and the growth occurs at times corresponding to the ballistic regime of particle motion (see Figure 3). Here, the particles move mainly along the vector of their initial velocity. Further, the value of the average linear displacement reaches its local maximum and, performing damped oscillations, reaches an almost constant value, where the diffusion mode of motion is realized. It can be seen that both the value of the first maximum and the constant value of saturation in the diffusion mode depend on the value of the average force $\bar{F}$. With increasing force values, the displacement increases, i.e., the activity of the system is increasing. Different values of the external force make it possible to realize different effective parameters of the nonideality of the system. For the value of the average linear displacement perpendicular to the vector of the initial velocity (inset in Figure 5), there is a slight increase in the amplitude of oscillations near zero.

**Figure 6:**
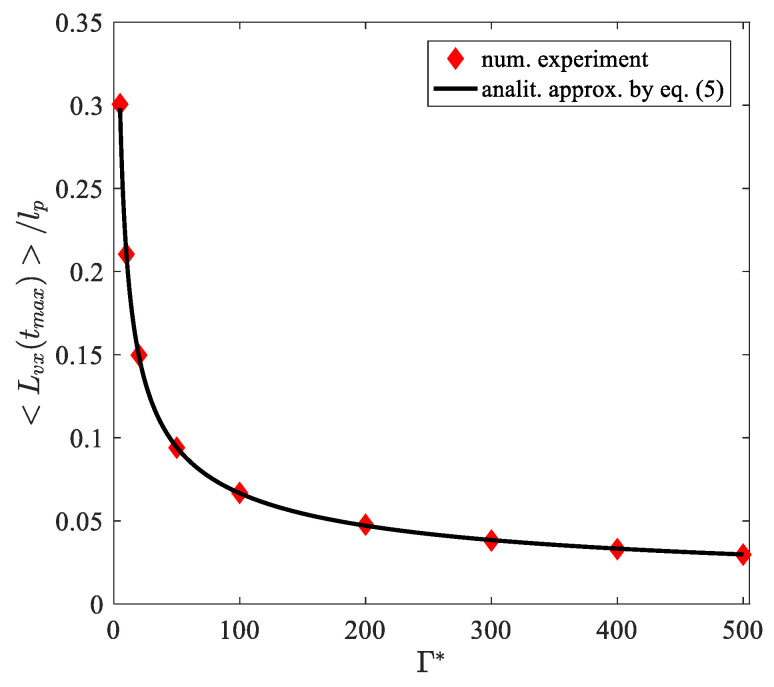
Dependence of the maximum values of the average linear displacements of active particles in a plasma-dust monolayer along the vectors of the initial velocities on the steady-state effective parameter of nonideality of the system.

Figure 6 shows the dependence of the maximum value of the average linear displacement along the vector of the initial velocity on the effective parameter of the nonideality of the system realized under the given conditions. It can be seen from the graph that the maximum values that the value $\langle L_{vx}\left(t_{\max}\;\right)\rangle$ takes is associated with the effective parameter of the system’s nonideality through a simple relation:

(5) $$\frac{\langle L_{vx}\left(t_{max}\right)\rangle }{l_{p}}\approx \frac{2}{3\sqrt{\Gamma^{*}}}.$$

Relation (5) can be used to determine the effective nonideality parameter of a system of active dust particles in a dust-plasma monolayer in a wide range with known experimental data on the coordinates and velocities of particles.

## 4. Conclusions

In this work, using the method of nonequilibrium Brownian dynamics, the microscopic dynamics of a system of interacting dust particles with a uniform metal coating under the action of laser radiation is investigated. Data on the coordinates and velocities of particles are obtained for different initial effective parameters of nonideality and different intensities of laser radiation. Using these data, the time dependences of the root-mean-square and average linear displacements of particles were calculated. It was found that a twofold increase in the average force caused by thermophoresis when the system is irradiated with a laser can lead to a fivefold decrease in the effective nonideality parameter. A simple relationship is established that connects the effective parameter of nonideality of the dusty plasma system of active particles with the maximum value of the mean linear displacement of particles along the direction of their initial velocities. This ratio can be used to diagnose such systems in a wide range of changes in the effective parameter of nonideality.

## Sample Availability

Samples of the compounds are not available from the authors.
